# Intraretinal changes in idiopathic versus diabetic epiretinal membranes after macular peeling

**DOI:** 10.1371/journal.pone.0197065

**Published:** 2018-05-08

**Authors:** Mario R. Romano, Gennaro Ilardi, Mariantonia Ferrara, Gilda Cennamo, Davide Allegrini, Pia Pafundi, Ciro Costagliola, Stefania Staibano, Giovanni Cennamo

**Affiliations:** 1 Department of Biomedical Sciences, Humanitas University, Rozzano, Milano, Italy; 2 Department of Biomedical Advanced Sciences, University Federico II, Naples, Italy; 3 Department of Neuroscience, Reproductive and Odontostomatological Science, Federico II University, Naples, Italy; 4 Department of Clinical and Experimental Medicine, University of Campania "Luigi Vanvitelli", Naples, Italy; 5 Eye Clinic, Department of Health Sciences, University of Molise, Campobasso, Italy; Massachusetts Eye & Ear Infirmary, Harvard Medical School, UNITED STATES

## Abstract

**Introduction:**

Epiretinal traction is not responsible only for epiretinal but also intraretinal changes. This study aims to describe structural and vascular intraretinal changes after macular peeling in idiopathic (iERM) vs diabetic ERM (dERM).

**Methods:**

We conducted a prospective interventional study on forty-two eyes, 23 with iERMs and 19 with dERMs, undergoing ERM-ILM peeling. We performed SD-OCT preoperatively, 1 and 6 months postoperatively to assess central macular thickness (CMT), intraretinal cysts (IC) and/or continuous ectopic inner foveal layers (CEIFL), superficial and deep capillary free zone (CFZ) area on OCT-A. Glial fibrillary acidic protein (GFAP), as a Müller cells marker, was detected immunohistochemically on ILM specimens, to assess Müller cells iatrogenic damage.

**Results:**

The CEIFLs were significantly more common in iERMs (12 (52.2%) in iERMs vs 2 (10.5%) in dERMs, p = 0.004), whereas ICs in dERMs (6 (26.1%) in iERMs vs 17 (89.5%) in dERMs, p<0.001). Median preoperative and postoperative BCVA was 20/50 [20/40-20/66] and 20/33 [20/25-20/40] in iERMs and 20/100 [20/66-20/200] and 20/50 [20/50-20/66] in dERMs, respectively. Median preoperative and postoperative CMT was 423 [370–488] and 364 [329–382] μm in iERM group and 465 [447–503] and 378 [359–433] μm in dERM group, respectively. The BCVA improvement and reduction of CMT thickness were significant in both groups (p<0.001). The presence of CEIFL was associated with lower BCVA in iERMs. Deep CFZ network significantly increased only in dERMs, passing from 0.34 [0.29–0.42] mm^2^ preoperatively to 0.56 [0.46–0.6] mm^2^ at 6-month follow-up (p<0.001). The GFAP expression was significantly higher in dERMs (p = 0.001).

**Conclusion:**

The intraretinal changes are different in iERMs and dERMs, as increased expression of CEIFLs in iERMs vs ICs in dERMs. The CEIFLs are associated with worse anatomical and functional outcomes in iERMs, whereas GFAP espression in peeled ILMs is higher in dERMs.

## Introduction

Epiretinal membranes (ERM) consist of non-vascularized fibrocellular tissue, formed by cellular metaplasia and proliferation at the vitreoretinal interface. In recent studies the prevalence varied from 1.02% to 26.1% [1–3]. The ERM is clinically classified as idiopathic (iERM), if no secondary causes are identified [4]. The ERM can be also secondary to the diabetic retinopathy (dERM); moreover, diabetic retinopathy is a risk factor for the development of secondary ERMs [2,3]. It is hypothesized that microscopic breaks of internal limiting membrane (ILM) following an anomalous posterior vitreous detachment (PVD) allow the migration of glial cells on the retinal surface and their subsequent proliferation with the interposition of the remnants of native vitreous between the ILM and the epiretinal tissue [5,6]. The Müller cells are the glial cells regulating retinal homeostasis and supporting structurally the foveola due to their binding of the photoreceptors; the footplates and the basal membrane of Müller cells form the ILM, preserving the retinal cytoarchitecture [7]. In response to retinal injuries or pathologies, such as ERM, and hyperglycemia there is an activation of Müller cells with reactive gliosis, characterized by hypertrophy, proliferation and upregulation of glial fibrillary acidic protein (GFAP) [8,9]; this intermediate filament strengthens the Müller cells-ILM bond, acting as a bridge, due to its interaction with cytoskeleton, surface receptors, and the proteins of extracellular matrix [10,11]. Although this process aimed to avoid neuroretinal degeneration, it causes epiretinal and intraretinal fibrosis [12,13]. It is conceivable that tangential centripetal traction and the overexpression of GFAP may cause intraretinal changes, such as outer and inner neuronal damage and structural foveal alterations [14]. Continuous ectopic inner foveal layers (CEIFL) have been recently described on OCT scans as an intraretinal uninterrupted band from inner retinal layers across the fovea [15]. On the other hand, different intraretinal changes have been reported in dERMs. These changes can be attributed to the early neuroretinal and microvascular impairment associated with diabetes [16].

Pars plana vitrectomy (PPV) with double peeling (ERM and ILM) is commonly employed by many surgeons for the removal of ERM [17]. However, it has been demonstrated that the ERM/ILM peeling induces the intraretinal collapse of Müller cells by damaging and removing their footplates [7,18]; the resulting glial apoptosis may cause the structural weakening of the retina [19]. Moreover, the risk of iatrogenic damage to the inner retina is increased when GFAP is overexpressed since it creates a strong the adhesion between ILM and Müller cells [7,11,18]. Since GFAP is marker of gliotic activation within Müller cells, its immunohistochemical detection on peeled tissue proves the structural injury of these cells induced by macular peeling [8,10].

The aim of this study is to investigate structural and vascular retinal changes following macular peeling in both iERMs and dERMs, comparing central macular thickness (CMT), presence of intraretinal cysts and CEIFL, superficial and deep capillary-free zone (CFZ) areas preoperatively (baseline) and 1 and 6 months after surgery. In addition, we performed the immunohistochemical analysis in order to evaluate the presence of GFAP on peeled ILMs.

## Materials and methods

### Study design

We conducted a prospective, interventional case series. The study adhered to the Declaration of Helsinki and was approved by the Institutional Review Board. All patients were enrolled in the Ophthalmic Unit of University of Naples “Federico II” and signed an informed consent before undergoing surgery by a single experienced surgeon (MRR). We included patients older than 18 years old affected by iERM or dERM, with disturbing metamorphopsia, best corrected visual acuity (BCVA) less than 0.22 logMAR (20/33) and clear evidence of tractional epiretinal membrane on sagittal and *en-face* OCT scans. We recruited patients that had indication for vitrectomy and ERM/ILM peeling and were waiting for surgery. The exclusion criteria were pregnancy, proliferative diabetic retinopathy, uncontrolled/untreated ocular diseases leading to significantly higher risk of complications during and after surgery, potential cause of visual impairment other than ERM, and severe systemic diseases that significantly increase the operative risk. On the basis of modified Early Treatment Diabetic Retinopathy Study (ETDRS) retinopathy severity scale all patients with dERMs were diagnosed with moderate non-proliferative diabetic retinopathy (NPDR) [20]; we excluded patients with intraretinal cysts larger than 400 μm, because of the subfoveal atrophy demonstrated in these patients after ILM peeling [7], patients with previous clinically significant diabetic macular edema (CMSE) as the fovea avascular zone (FAZ) is enlarged in this subgroup [21] and patients treated in the last 6 months with intravitreal anti-vascular endothelial growth factor (anti-VEGF) injections or laser photocoagulation.

### Participants

Forty-two patients, 23 affected by iERM and 19 by dERM, underwent double (ERM/ILM) macular peeling between June and August 2016. The clinical ocular examination was conducted before, 1 and 6 months after surgery, including BCVA test, slit-lamp biomicroscopy, dilated funduscopy, and SD-OCT scan with OCT-A. The BCVA was measured on a Snellen chart. The SD-OCT was performed by one of two masked physicians (MF or GlC) to evaluate CMT and the presence of CEIFLs and intraretinal cysts between 50 and 400 μm; superficial and deep capillary free zone (CFZ) area were analyzed on OCT-A scans preoperatively, 1 and 6 months after surgery.

### Spectral domain-OCT scan protocol and analysis

We used the RTVue XR Avanti (Optovue Inc., Fremont, CA, USA). The images were considered suitable if signal strength index was ≥50. The measurements of MT were at the fovea, parafovea and perifovea within 1, 1–3, and 3–5 mm from the foveal center, respectively, and the mean CMT was assessed automatically through the “retina map” function of the RTVue XR Avanti. The macula raster and cross line scanning with 21 B-scans over 12x4mm area was performed in all eyes; the CEFILs were identified as defined by Govetto et al. on the SD-OCT fovea-centered scans and manually measured using the caliper.

To acquire OCT-A data, we used the AngioVue software of RTVue XR Avanti with the SSADA algorithm and the A-scan rate of 70.000 scans per second. A central wavelength of 849 nm was covered by the scans with 5μm of tissue axial depth resolution. We used 3×3 mm fovea-centered scanning area. The size of superficial and deep CFZ areas was automatically measured by the software, manually modified in case of inaccuracy in the borders.

### Surgical technique

A trans-conjunctival sutureless PPV was performed in all patients with the Constellation 25 G+ Total Plus Vitrectomy Pak (Alcon Laboratories, Inc, Fort Worth, TX). The core vitrectomy was carried out, inducing a complete PVD by aspiration. The dye injected was trypan blue 0.15% + brilliant blue G 0.025% + 4% polyethylene glycol (Membrane-Blue-Dual, DORC International, The Netherlands). Fine-tipped forceps (Alcon ILM forceps 25G) were used to peel the ERM and then the ILM. The periphery was checked with scleral indentation and a fluid-air exchange was performed at the end. A combined phacovitrectomy was performed in all phakic patients.

### Immunohistochemical evaluation

All formalin-fixed paraffin-embedded ILM specimens were cut in 5-μm-thick serial sections. After the confirmation of the sample’s adequacy by one hematoxylin/eosin-stained section, the following section was processed for immunohistochemistry (IHC), mounted on glass slides coated with poly-L-lysine. We used an automated IHC System (Ventana BenchMark XT; Ventana Medical Systems), incubating ILM sections with antibodies against GFAP (rabbit monoclonal, clone EP672Y, Ventana Medical Systems) as markers of glial cells. Mayer’s hematoxylin was used to counterstain all sections. Two blinded observers (SS and GI) evaluated independently the expression of GFAP on ILM slides, resolving by consensus controversial cases. They scored the intensity of the staining from 0 to 3 (0 = absent; 1 = mild; 2 = moderate; 3 = intense), as well as the percentage of stained cells (0 = 0; 1 = 10%; 2 = 10–25%; 3 = 25%). The values assigned were then summed to obtain a semi-quantitative score (H-score).

### Statistical analysis

All Snellen BVCA values were converted into logarithm of the minimal angle of resolution (logMAR) units to perform the statistical analysis. The continuous or numeric variables were presented as median and interquartile range (IQR), whilst the categorical variables as number and percentage. The statistical significance of the differences observed with respect to the presence or absence of mutation was evaluated by either Chi-squared test or Fisher exact test (in the case of a low number of one of the groups, n < 50) for categorical variables and the Mann-Whitney U test or Kruskal-Wallis test for numeric variables, respectively for two or multiple independent samples, whilst the test of Wilcoxon was used for paired samples (i.e., repeated measures). A value of p ≤ 0.05 was taken as an indication of the statistical significance of observed differences. All analyses were performed with SPSS 24 software, with a 5% significance level and two-sided test.

## Results

Twenty-three patients affected by iERM and 19 by dERM underwent PPV and ERM/ILM peeling. All baseline demographic and clinical data are shown in Table 1 (S1 PDF); no statistically significant difference was found regarding age, gender, eye, lens status and mean duration of symptoms before surgery between the two groups; whereas, CEIFLs were significantly more common in iERM than dERM group, contrary to presence of intraretinal cysts (Table 1). We adopted the staging system proposed by Govetto et al [15], that divided ERMs on the basis of some OCT findings, such as presence of foveal pit (stage 1), absence of foveal pit and CEIFLs (stage 2), presence of CEIFLs without (stage 3) or with (stage 4) disruption of retinal layers. At baseline we found in iERM group the stage 2 ERMs in 10 patients (43.48%), stage 3 in 11 (47.83%) and stage 4 in 2 (8,69%); whereas in dERM group 17 patients with stage 2 ERM (89.47%), 1 with stage 3 (6.25%) and 1 with stage 4 (6.25%). Due to the paucity of diabetic patients with CEIFL, we reported the mean CEIFL thickness values only for iERM group (Table 2), after excluding one patient with stage 4 iERM in which the markedly altered retinal architecture did not allow the measurement of CEIFL thickness.

**Table 1:** Demographic anf clinical findings at baseline.

Parameter	iERM (n = 23)	dERM (n = 19)	p-value
**Age (years), median [IQR]**	67 [66–69]	68 [65–72]	0.761
**Male (%)/Female (%)**	9 (39.1)/14 (60.9)	7 (36.8)/12 (63.2)	1.000
**Right eye (%)/ Left eye (%)**	13 (56.5)/10 (43.5)	13 (68.4)/6 (31.6)	0.530
**Phakic (%)/Pseudophakic (%)**	17 (73.9)/6 (26.1)	15 (78.9)/4 (21.1)	1.000
**Duration of symptoms (months), median**	8 [7–10]	9 [7–11]	0.385
**BCVA (logMAR), median [IQR]**	0.4 [0.3–0.5]	0.7 [0.5–1]	0.000
**MT (μm), median [IQR]**	423 [370–488]	465 [447–503]	0.077
**Presence/Absence of CEIFL (%)**	12 (52.2)/11 (47.8)	2 (10–5)/17 (89.5)	0.004
**Presence/Absence of intraretinal cysts (%)**	6 (26.1)/17 (73.9)	17 (89.5)/2 (10.5)	0.000
**Sup CFZ (mm**^**2**^**), median [IQR]**	0.26 [0.15–0.31]	0.17 [0.13–0.25]	0.065
**Deep CFZ (mm**^**2**^**), median [IQR]**	0.22 [0.19–0.3]	0.34 [0.29–0.42]	0.001

iERM: idiopathic epiretinal membrane; dERM: diabetic epiretinal membrane; IQR: interquartile range; BCVA: best corrected visual acuity; logMAR: logarithm of the minimal angle of resolution; CEIFL: continuous ectopic inner foveal layers; CFZ: capillary free zone.

**Table 2:** Clinical findings preoperatively, 1 and 6 months after macular peeling in idiopathic ERMs.

Parameter	Pre	1m follow-up	6m follow-up	p (pre vs 1m)	p (pre vs 6m)	p (1m vs 6m)
**BCVA (logMAR), median [IQR**^**c**^**]**	0.4 [0.3–0.5]	0.3 [0.22–0.3]	0.22 [0.1–0.3]	0.000	0.000	0.001
**CMT (μm), median [IQR]**	423 [370–488]	384 [344–403]	364 [329–382]	0.000	0.000	0.000
**CEIFL thickness (μm), median [IQR]**	161 [113.25–250]	106.5 [80.5–158.5]	82 [73–100]	0.002	0.005	0.005
**Sup CFZ (mm**^**2**^**), median [IQR]**	0.26 [0.15–0.31]	0.21 [0.12–0.31]	0.22 [0.15–0.3]	0.601	0.944	0.904
**Deep CFZ (mm**^**2**^**), median [IQR]**	0.22 [0.19–0.3]	0.23 [0.19–0.29]	0.22 [0.19–0.26]	0.711	0.664	1.000

BCVA: best corrected visual acuity; logMAR: logarithm of the minimal angle of resolution; IQR: interquartile range; CMT: central macular thickness; CEIFL: continuous ectopic inner foveal layers; CFZ: capillary free zone.

The preoperative and postoperative findings of each group are shown in the Tables 2 and 3, respectively.

**Table 3:** Clinical findings preoperatively, 1 and 6 months after macular peeling in diabetic ERMs.

Parameter	Pre	1m follow-up	6m follow-up	p (pre vs 1m)	p (pre vs 6m)	p (1m vs 6m)
**BCVA (logMAR), median [IQR**^**c**^**]**	0.7 [0.5–1]	0.5 [0.4–0.7]	0.4 [0.4–0.5]	0.008	0.002	0.157
**CMT (μm), median [IQR]**	465 [447–503]	400 [385–456]	378 [359–433]	0.001	0.000	0.000
**Sup CFZ (mm**^**2**^**), median [IQR]**	0.17 [0.13–0.25]	0.18 [0.14–0.24]	0.18 [0.16–0.23]	0.337	0.194	0.717
**Deep CFZ (mm**^**2**^**), median [IQR]**	0.34 [0.29–0.42]	0.48 [0.42–0.55]	0.56 [0.46–0.6]	0.000	0.000	0.000

BCVA: best corrected visual acuity; logMAR: logarithm of the minimal angle of resolution; IQR: interquartile range; CMT: central maculat thickness; CFZ: capillary free zone.

### Visual acuity measures

Best corrected VA significantly improved at 1 and 6 months after surgery (Tables 2 and 3) (S1 PDF). In iERM group we compared separately BCVA in stage 2 and stage 3 ERMs. In stage 2 iERMs the median BCVA was 20/40 [20/33-20/50] at baseline, 20/33 [20/29-20/35] at 1-month follow-up (f/u) and 20/25 [20/22-20/33] at 6-month f/u, whereas in stage 3 iERMs BCVA was 20/50 [20/50-20/66], 20/40 [20/33-20/40] and 20/33 [20/29-20/40] preoperatively, 1 and 6 months after surgery, respectively. Therefore, the preoperative presence of CEIFL (stage 3 ERMs) was associated with lower BCVA both preoperatively (p = 0.024) and 1 month (p = 0.008) and 6 months (p = 0.005) after surgery, compared to stage 1 and 2 ERMs (absence of CEIFL). We did not evaluate the same association in dERM group because the number of patients with CEIFL was too small to perform any statically significant analysis.

### OCT findings

In both groups there was a statistically significant reduction in CMT at both 1- and 6-month f/u visit (Tables 2 and 3). In iERM group CEIFLs disappeared in 2 cases (15.4%), whereas in all the remaining cases CEIFL thickness was significantly reduced (Table 2). Of the two patients with dERM and CEIFLs, the latter persisted in one of them (50%) after surgery.

The postoperative disappearance of intraretinal cysts was observed in 5 of 6 iERMs (83.3%) and in 8 of 17 dERMs (47.1%).

**Fig 1:**
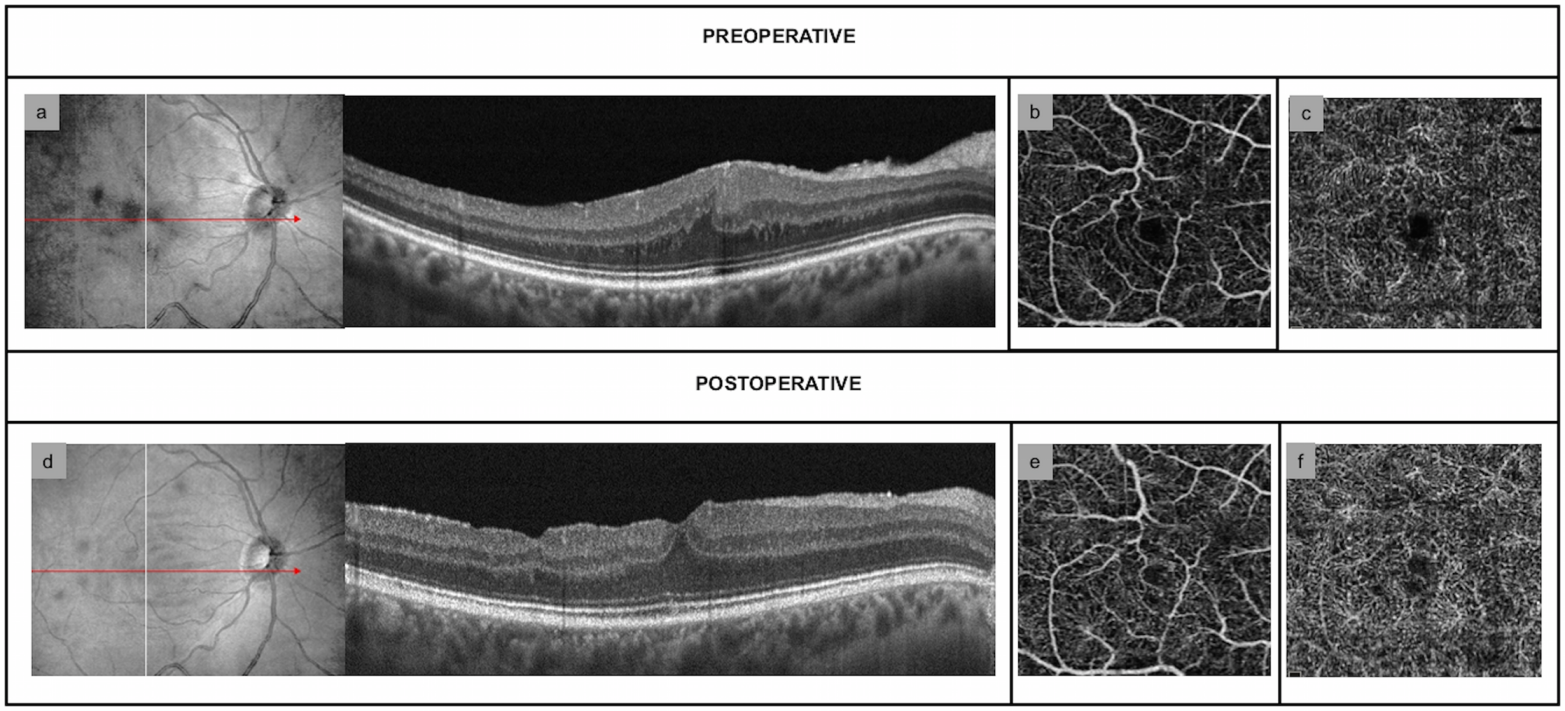
Structural and vascular findings in idiopathic epiretinal membranes. (a) Preoperative OCT-scan showing the presence of continuous ectopic inner foveal layers (CEIFLs) (b) Preoperative OCT-angiography (OCT-A) scan of superficial capillary free zone (CFZ) (c) Preoperative OCT-A scan of deep capillary free zone (d) OCT-scan 6 months after macular peeling. The central macular thickness is reduced and the CEIFLs disappeared (e) 6-month OCT-A scan of superficial CFZ without any significant changes of superficial capillary free zone area 6 months after surgery (f) Deep CFZ 6 months after surgery: no significant changes are detectable.

**Fig 2:**
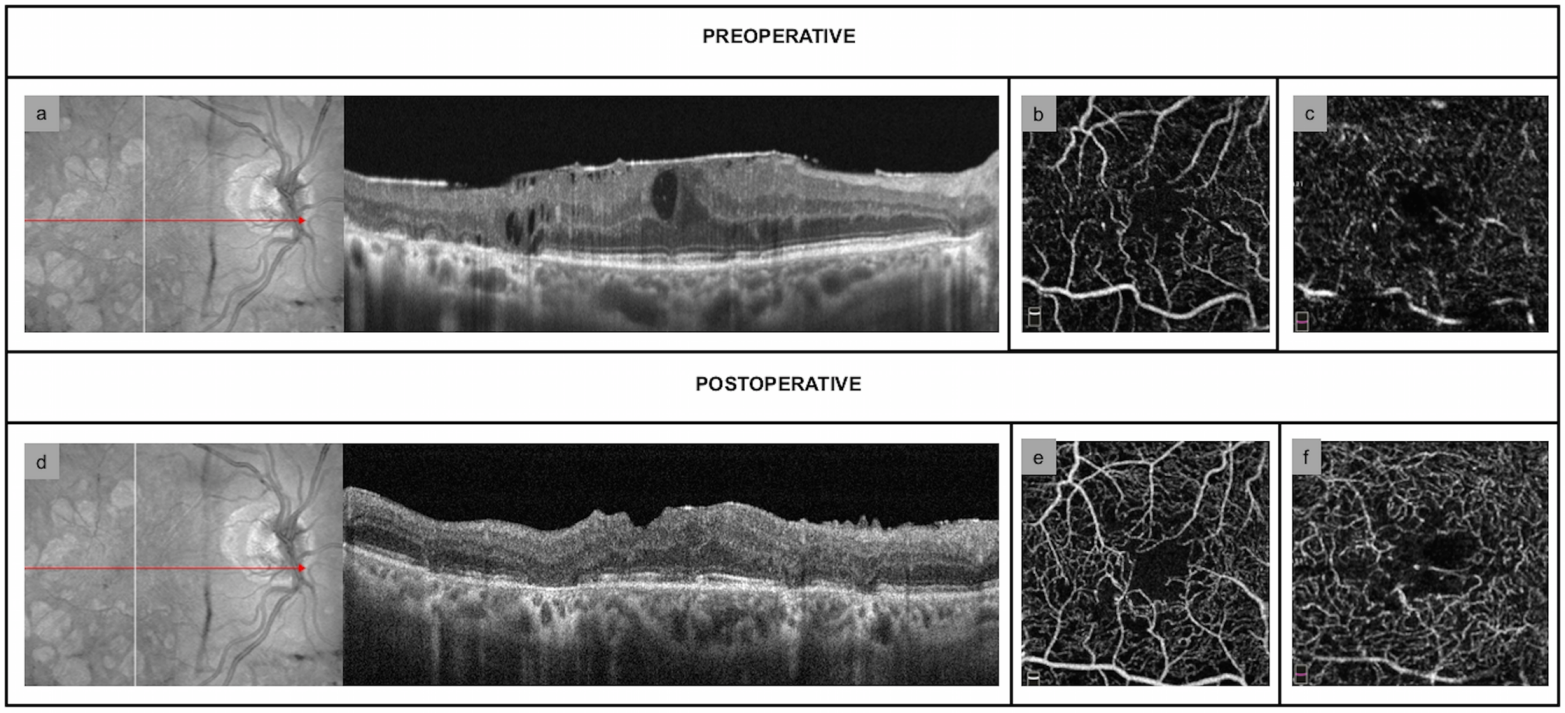
Structural and vascular findings in diabetic epiretinal membranes. (a) Preoperative OCT-scan showing the presence of multiple intraretinal cysts (b) Preoperative OCT-angiography (OCT-A) scan of superficial capillary free zone (CFZ) (c) Preoperative OCT-A scan of deep CFZ (d) OCT-scan 6 months after macular peeling, showing the significant reduction in central macular thickness and the disappearance of the intraretinal cysts (e) OCT-A 6 months after surgery shows no significant changes of superficial CFZ (f) 6-month OCT-A scan of deep CFZ, that appears enlarged.

The difference between preoperative and postoperative superficial CFZ area was not statistically significant in both groups, whereas the deep CFZ network was significantly increased only in dERMs at both 1- and 6-month follow up visit (*P*<0.0001) (Tables 2 and 3) (Figs 1 and 2).

### Immunohistochemical analysis

The immunohistochemical analysis detected GFAP in 21 of 23 (91.3%) specimens in iERM group and in all specimens in dERM group. The results are resumed in Table 4 (S1 PDF). The H score was significantly greater in diabetic specimens than in idiopathic ones (*P* = 0.001).

**Table 4:** Immunohistochemical results on internal limiting membranes peeled.

GFAP	iERM (n = 23)	dERM (n = 19)	p-value
% stained cells	1 [1–2]	2 [2–3]	0.004
Stain intensity	2 [1–2]	2 [2–3]	0.022
H Score	3 [2–4]	5 [4–5]	0.001

GFAP: glial fibrillary acid protein, iERM: idiopathic epiretinal membrane, dERM: diabetic epiretinal memebrane.

## Discussion

Pars plana vitrectomy with double (ERM and ILM) macular peeling is a common procedure for symptomatic patients affected by ERM [17]. The mechanical trauma that ILM peeling induces on Müller cells damaging their basement membrane has been supported by finding of Müller cell debris and retinal tissue on peeled ILM specimens [22]. We investigated structural and vascular intraretinal changes following macular peeling in both iERMs and dERMs.

At baseline, we found that the presence of CEIFLs was significantly different in the two groups accounting for 13 eyes (52.2%) in iERM group and 2 eyes (10.5%) in dERM group, respectively (P = 0.004); on the contrary, intraretinal cysts were significantly more common in dERMs (P<0.0001). We believe that this finding could be due to different status of Müller cells in iERM versus dERM patients. The chronic tractional stress exerted by iERM could mechanically displace the inner retinal layers centripetally. In addition, it also acts on healthy Müller cells inducing their activation with over-expression of GFAP and reactive intraretinal gliosis. As we already stated above, the increased GFAP, ubiquitous and unspecific response of Müller cells to retinal pathologies and injury, may create a strong adhesion between Müller cells and both ILM and epiretinal tissue. Moreover, the inner processes of the foveolar Müller cells form a network extending horizontally that could be involved in the alteration of the foveolar shape [23]. The combination of intraretinal gliosis and physical traction could result in the formation of CEIFLs [15]. On the contrary, it has been documented that in diabetic retinopathy there are an inner neuroretinal degeneration prior to micro-vasculopathy [24] and a high glucose (HG)-induced mitochondrial dysfunction that promotes the apoptosis of Müller cells, compromising their protective role towards neurons [25]. On the other side, since the Müller cells play an important role in the maintenance of the inner blood retinal barrier (BRB) and in retinal VEGF production [26], this can contribute to increase vascular permeability. We hypotize that in dEMRs the injury and loss of Müller cells may result, under traction, in less reactive intraretinal gliosis with consequent absence of CEIFL, whereas end up in the greater percentage of intraretinal cysts.

Structural outcomes have been well reported in literature in terms of reduced macular thickness after ERM/ILM peeling in patients affected by iERMs [11,27]. We found a statistically significant reduction of CMT in both groups. In 11 of 13 iERMs (84.6%) CEIFL persisted after surgery with a significant reduction in thickness, consistently with a recent study [28].

Regarding vascular changes after macular peeling, we found a statistically significant postoperative enlargement of deep CFZ network only in diabetic patients, in accordance with a previous study [11]. In diabetic patients the microcirculation, impaired by hyperglycemia, may be further damaged by the upregulation of GFAP with the subsequent gliosis and the ILM peeling with Müller cells collapse [7,10]. Recent studies showed that in diabetic eyes, even in absence of retinopathy, the macular microcirculation is impaired, the foveal avascular zone is larger than in healthy ones, and the perifoveal intercapillary area increases directly with the progression of diabetic retinopathy [21,29,30]. The neurodegeneration seems also to contribute to early diabetic microvascular changes [31]. In eyes with diabetic retinopathy dilated capillaries, intraretinal hemorrhages, microaneurysms, alterated blood retinal barrier, non-perfused areas, vasoregression, capillary closure and the subsequent abnormal anastomoses and shunts lead to impair the perifoveal capillary plexus. All the above mentioned conditions may make macula and perifoveal capillary plexus more sensitive to the mechanical damage of Müller cells induced by ILM peeling [11]. The consequent deep capillary ischemia and macular non-perfusion contribute to disrupt the outer retina [32]. We are not able to explain the sparing of superficial capillary plexus although it is directly exposed to the mechanical stretching; however, it has been reported that in diabetic retinopathy changes in terms of enlarged FAZ in deep capillary plexus are more common than in superficial one [33,34].

Functionally, 1-month and 6-month BCVA was significantly improved in both groups, consistently with previous studies [17]. Indeed, it has been reported that the mechanical ILM-peeling-induced Müller cells damage and the consequent postoperative ultrastructural changes do not significantly influence BVCA in iERMs, whereas lead to subtle functional alterations detectable only through measures more sensitive than BCVA, such as microperimetry and electroretinogram [18,22,35]. Minnella et al. also reported the absence of significant correlation between VA and perifoveal vascular areas in diabetic patients with macular ischemia [36]. The preoperative presence of CEIFL has been independently and significantly associated with lower BCVA at baseline and 1 year after ERM/ILM peeling in iERMs [15,28]. In order to verify the influence of CEIFL on BCVA, we compared the latter between stage 2 and stage 3 iERMs, excluding stage 4 iERMs due to the small number of eyes. Albeit limited by the sample size, our data confirmed the results of the previous studies [15,28]. As already proposed by Govetto et al [15], we believed that this association could be due to the combination of two factors: on one side, the physical traction on retinal layers with obstruction between the afferent light and the foveal photoreceptors, displacement and deformation of the photoreceptors alterating neurotransmission; on the other side, the reactive proliferation driven by Müller cells. Moreover, the lower preoperative BCVA is a negative functional prognostic factor itself [37]. The residual chronic alteration of foveal microanatomy in combination with the reactive reaction of Müller cells formation of bridge of retinal tissue in in the foveal area has been supposed to play a role in the lower postoperative functional recovery of patient with CEIFLs [28].

We have also evaluated the presence of GFAP in the ILMs peeled. We found a significantly greater expression of GFAP on ILM specimens of dERM group (p<0,05). It could be argued that the various degrees of staining in peeled ILMs could represent the variability of retinal response to the disease and cellular migration and that we previously mentioned a minor reactive gliosis in dERMs. However, we believe that this finding may be explained on the basis of the peculiar features of diabetic ILM. In addition to ERM, also hyperglycemic conditions and diabetic retinopathy are known to induce the further activation of the Müller cells and the over-expression of GFAP [11]. The advanced glycation end-products in the posterior vitreous cortex with cross-linking of collagen fibrils lead to a stronger adhesion between ILM and vitreous remnants in patients with diabetic macular edema (DME) [7]. Moreover, in patients with ERM and DME the proliferation of inflammatory cells, such as macrophages, neutrophils, lymphocytes, fibroblast-like cells and glial cells, on the vitreous side of the ILM make it thicker [38].

These data suggest that in patients with dERMs due to the underlying pathological retinal condition, on one side, the macula is more sensitive to the peeling-induced damage and, on the other side, the peeling lead to a greater injury in presence of a thicker and more rigid ILM, with consequent outer retina damage and retinal structural collapse. The reduction of macular volume in diabetic patients has also been associated with a contraction of Müller cells, induced by ILM peeling [7].

We acknowledged that the small sample size and the short follow-up were limitations of this study. Moreover, regarding of OCT protocol, the manual measurements may result in errors and the need of precise fixation is an intrinsic limitation of OCT-A scans. On the other hand, the strength of this study is the prospective nature, except for CEFIL data that have been retrospectively collected, and the immunohistochemical evaluation of the peeled ILMs.

In conclusion, we suggest that the different underlying status of Müller cells in iERMs and dERMs lead to different intraretinal changes, such as the presence of CEIFL in iERM and intraretinal cysts in dERMs. In iERMs, for timing and planning of macular peeling, it should be considered that the presence of CEIFL is associated with worse functional and anatomical recovery, although a significant BCVA improvement has been largely documented. Moreover, assuming the established damage that ILM peeling induces on Müller cells, in dERM, where the Müller cells are already impaired, the surgical trauma may result in even greater injury.

## Supporting information

S1 PDF Data of study patients. Demographic and preoperative and postoperative clinical finidngs of patients affected by idiopathic and diabetic ERMs.
(PDF)
