# Correction: Intraretinal changes in idiopathic versus diabetic epiretinal membranes after macular peeling

**DOI:** 10.1371/journal.pone.0201503

**Published:** 2018-07-25

**Authors:** Mario R. Romano, Gennaro Ilardi, Mariantonia Ferrara, Gilda Cennamo, Davide Allegrini, Pia Clara Pafundi, Ciro Costagliola, Stefania Staibano, Giovanni Cennamo

The sixth author’s name appears incorrectly. The correct name is: Pia Clara Pafundi. The correct citation is: Romano MR, Ilardi G, Ferrara M, Cennamo G, Allegrini D, Pafundi PC, et al. (2018) Intraretinal changes in idiopathic versus diabetic epiretinal membranes after macular peeling. PLoS ONE 13(5): e0197065. https://doi.org/10.1371/journal.pone.0197065
