# Supplementary material for: Intraretinal changes in idiopathic versus diabetic epiretinal membranes after macular peeling
Source: PLoS One. 2018 May 8;13(5):e0197065. doi: 10.1371/journal.pone.0197065 (PMC5940191; doi:10.1371/journal.pone.0197065)
Supplement: S1 PDF — Demographic and preoperative and postoperative clinical finidngs of patients affected by idiopathic and diabetic ERMs. (PDF) [file pone.0197065.s001.pdf]

Pt iERM	Age	Eye	Sex	Lens	Sympt	BVCA pre	BVCA 1m	BVCA 6m	stage pre	CEIFL pre	CEIFL 1m	CEIFL 6m	disap CEIFL	IR cysts	disap IR cysts	MT pre	MT 1m	MT 6m	sup CFZ pre	sup CFZ 1m	sup CFZ 6m	deep CFZ pre	deep CFZ 1m	deep CFZ 6m	GFAP cell	GFAP int	H-GFAP
1	67	1	1	0	12	0.22	0.16	0	2					1	1	300	295	280	0.4	0.38	0.39	0.25	0.25	0.24	1	1	2
2	65	1	1	0	7	0.3	0.22	0.1	2					1	1	370	356	336	0.05	0.07	0.07	0.47	0.44	0.46	1	1	2
3	80	1	1	0	6	0.5	0.22	0.16	2					1	1	456	408	367	0.31	0.43	0.45	0.09	0.07	0.08	2	2	4
4	76	1	1	0	7	0.4	0.3	0.1	2					0		378	354	296	0.27	0.27	0.29	0.32	0.26	0.21	1	1	2
5	66	1	1	0	5	0.22	0.16	0.05	2			N/A		0		276	262	284	0.26	0.25	0.22	0.34	0.3	0.25	1	1	2
6	64	1	1	0	11	0.4	0.22	0.22	2					0		423	375	354	0.19	0.19	0.26	0.22	0.32	0.36	1	1	2
7	67	1	1	0	11	0.3	0.3	0.22	2					0		398	343	329	0.13	0.12	0.15	0.17	0.33	0.36	1	1	2
8	74	1	1	0	10	0.3	0.16	0.1	2					0		362	396	367	0.27	0.31	0.33	0.19	0.19	0.27	0	0	0
9	59	1	1	0	8	0.22	0.1	0.05	2					0		352	339	334	0.22	0.15	0.15	0.28	0.23	0.19	0	0	0
10	63	1	1	0	7	0.3	0.22	0.22	2					0		372	344	328	0.21	0.21	0.19	0.23	0.23	0.19	1	2	3
11	69	0	1	0	14	0.3	0.22	0.16	3	84	47	N/A	1	0		382	366	354	0.3	0.12	0.09	0.21	0.18	0.16	1	1	2
12	67	0	1	0	9	0.3	0.22	0.3	3	95	45		1	0	N/A	344	338	309	0.32	0.31	0.28	0.22	0.17	0.1	2	1	3
13	68	0	1	0	8	0.4	0.3	0.22	3	135	94	67	0	0		437	385	376	0.33	0.33	0.31	0.35	0.34	0.2	2	2	4
14	67	0	1	0	8	0.4	0.3	0.16	3	164	115	99	0	0		488	403	384	0.39	0.38	0.38	0.16	0.21	0.22	3	3	6
15	68	0	0	0	6	0.4	0.3	0.16	3	138	98	83	0	0		467	384	367	0.12	0.19	0.21	0.24	0.19	0.21	2	2	4
16	69	0	0	0	11	0.4	0.3	0.22	3	244	145	79	0	0		484	387	364	0.35	0.25	0.2	0.2	0.21	0.25	2	2	4
17	68	0	0	0	7	0.5	0.4	0.3	3	266	163	96	0	0		515	405	382	0.09	0.11	0.13	0.19	0.29	0.31	1	2	3
18	69	0	0	1	9	0,7	0.4	0.4	3	247	185	103	0	0		525	456	404	0.17	0.11	0.11	0.16	0.22	0.23	2	2	4
19	63	0	0	1	9	0.4	0.22	0.22	3	158	96	75	0	0		452	400	383	0.25	0.26	0.28	0.38	0.17	0.19	1	3	4
20	67	0	0	1	10	0.5	0.3	0.3	3	251	132	81	0	0		511	399	365	0.27	0.21	0.18	0.24	0.21	0.22	2	3	5
21	68	0	0	1	8	0.4	0.3	0.22	3	106	76	54	0	1	1	406	354	342	0.09	0.12	0.22	0.22	0.24	0.26	1	3	4
22	71	0	0	1	8	0.7	0.4	0.4	4	263	174	132	0	1	1	545	433	405				N/A			3	2	5
23	66	0	0	1	9	1	0.5	0.4	4		N/A		0	1	0	599	476	442							2	1	3

Pt dERM	Age	Eye	Sex	Lens	ymptom	BVCA pre	BVCA 1m	BVCA 6m	stage pre	CEIFL pre	CEIFL 1m	CEIFL 6m	ppearance C	IR cysts	ppearance IR c	MT pre	MT 1m	MT 6m	sup CFZ pre	sup CFZ 1m	sup CFZ 6m	deep CFZ pre	deep CFZ 1m	deep CFZ 6m	GFAP cell	GFAP int	H-GFAP
1	66	0	1	0	14	0.7	0	0.4	2					1	0	520	460	450	0.36	0.37	0.4	0.31	0.45	0.51	1	1	2
2	67	0	1	0	12	0.7	0.5	0.4	2					1	0	450	400	360	0.05	0.06	0.05	0.56	0.74	0.83	3	3	6
3	59	0	1	0	9	1	0.5	0.5	2					1	0	447	396	378	0.21	0.22	0.2	0.36	0.51	0.53	1	1	2
4	75	0	1	0	9	1	0.7	0.5	2					1	0	503	363	357	0.17	0.21	0.22	0.31	0.39	0.45	1	1	2
5	72	0	1	0	7	0.7	0.4	0.4	2					1	0	488	387	367	0.16	0.17	0.15	0.12	0.41	0.43	2	1	3
6	68	0	1	0	9	1	0.7	0.7	2					1	1	503	377	344	0.19	0.17	0.17	0.42	0.52	0.58	2	3	5
7	62	0	1	0	11	1	0.7	0.5	2					1	1	466	398	362	0.13	0.12	0.19	0.48	0.59	0.63	2	3	5
8	69	0	1	0	15	0.7	0.5	0.3	2					1	1	425	400	390	0.18	0.33	0.35	0.29	0.39	0.45	2	3	5
9	56	0	1	0	5	0.5	0.5	0.7	2			N/A		1	1	447	456	433	0.12	0.15	0.13	0.37	0.43	0.46	3	3	6
10	74	0	1	0	7	0.5	0.7	0.4	2					1	1	465	432	411	0.21	0.2	0.18	0.34	0.42	0.57	2	3	5
11	77	0	1	0	10	0.4	0.7	0.5	2					0	N/A	372	467	434	0.3	0.28	0.26	0.34	0.65	0.72	2	3	5
12	81	0	1	0	7	0.5	0.4	0.5	2					1	1	467	392	367	0.26	0.27	0.26	0.49	0.53	0.56	2	3	5
13	66	0	0	0	9	0.4	0.4	0.4	2					1	0	455	412	382	0.13	0.13	0.17	0.42	0.55	0.59	1	3	4
14	69	1	0	0	10	0.5	0.22	0.3	2					1	0	447	385	342	0.25	0.22	0.21	0.37	0.45	0.49	3	2	5
15	68	1	0	0	10	0.7	0.3	0.22	2					1	0	449	418	415	0.12	0.15	0.17	0.29	0.34	0.58	3	2	5
16	71	1	0	1	11	0.5	0.4	0.4	2					1	1	476	345	327	0.25	0.23	0.21	0.28	0.54	0.67	3	2	5
17	56	1	0	1	7	0.5	0.5	0.3	2					1	1	407	345	359	0.04	0.11	0.12	0.28	0.42	0.43	3	2	5
18	65	1	0	1	10	0.5	0.5	0.5	3	95	43	0	1	0	N/A	554	456	437	0.17	0.16	0.18	0.33	0.57	0.59	3	2	5
19	68	1	0	1	6	1.3	1	0.7	4		N/A		0	1	0	607	509	474	N/A	N/A	N/A	N/A	N/A	N/A	3	2	5
